# Silica nanoparticles with encapsulated DNA (SPED) – a novel surrogate tracer for microbial transmission in healthcare

**DOI:** 10.1186/s13756-020-00813-7

**Published:** 2020-09-16

**Authors:** Manuela Scotoni, Julian Koch, Timothy R. Julian, Lauren Clack, Ana K. Pitol, Aline Wolfensberger, Robert N. Grass, Hugo Sax

**Affiliations:** 1 Department of Infectious Diseases and Hospital Epidemiology, University Hospital Zurich, University of Zurich, Raemistrasse 100, HAL14, 8091 Zurich, Switzerland; 2 Department of Chemistry and Applied Biosciences, ETH Zurich, Zurich, Switzerland; 3 Department of Environmental Microbiology, Eawag, Swiss Federal Institute of Aquatic Science and Technology, Duebendorf, Switzerland

**Keywords:** Healthcare-acquired infection, Infection prevention, Infection control, Transmission, Bacterial transmission, Cross-infection, Cross-contamination, Nanoparticles, Tracers, DNA

## Abstract

**Background:**

The increase in antimicrobial resistance is of worldwide concern. Surrogate tracers attempt to simulate microbial transmission by avoiding the infectious risks associated with live organisms. We evaluated silica nanoparticles with encapsulated DNA (SPED) as a new promising surrogate tracer in healthcare.

**Methods:**

SPED and *Escherichia coli* were used to implement three experiments in simulation rooms and a microbiology laboratory in 2017–2018. Experiment 1 investigated the transmission behaviour of SPED in a predefined simulated patient-care scenario. SPED marked with 3 different DNA sequences (SPED1-SPED3) were introduced at 3 different points of the consecutive 13 touch sites of a patient-care scenario that was repeated 3 times, resulting in a total of 288 values. Experiment 2 evaluated SPED behaviour following hand cleaning with water and soap and alcohol-based handrub. Experiment 3 compared transfer dynamics of SPED versus *E. coli* in a laboratory using a gloved finger touching two consecutive sites on a laminate surface after a first purposefully contaminated site.

**Results:**

Experiment 1: SPED adhesiveness on bare skin after a hand-to-surface exposure was high, leading to a dissemination of SPED1–3 on all consecutive surface materials with a trend of decreasing recovery rates, also reflecting touching patterns in concordance with contaminated fingers versus palms. Experiment 2: Hand washing with soap and water resulted in a SPED reduction of 96%, whereas hand disinfection led to dispersal of SPED from the palm to the back of the hand. Experiment 3: SPED and *E. coli* concentration decreased in parallel with each transmission step – with SPED showing a trend for less reduction and variability.

**Conclusions:**

SPED represent a convenient and safe instrument to simulate pathogen spread by contact transmission simultaneously from an infinite number of sites. They can be further developed as a central asset for successful infection prevention in healthcare.

## Background

The increasing spread of antimicrobial resistance and the burden of healthcare-associated infections represent substantial threats to patient health and life [1–3]. Healthcare workers’ (HCW) hands play a key role in transmission of pathogens through bi-directional exchange of microbes between hands and surfaces, [4, 5] especially considering that hand hygiene performance is commonly substandard in almost all healthcare settings despite widely accepted concepts and guidelines [6, 7]. A recent head-camera-aided study uncovered that many more opportunities for microbe transmission might exist in acute healthcare than anticipated based on more traditional hand hygiene observation methods [8–10]. Tracing events leading to microbial transmission, however, is difficult, especially using living microorganisms. Surrogate tracers may therefore provide a safer and easier means to increase our understanding of transmission pathways. Prior studies have attempted to trace transmission events using microbial surrogates, [11–16] and/or DNA [11, 17–21]. Use of microbial surrogates is limited to benign organisms, like MS2 phage, and therefore limits their widespread use in transmission studies. DNA transmission behaviour is unlikely to mimic that of microorganisms. Cauliflower mosaic virus DNA, [11, 17–21] bacteriophages, and fluorescent lotion have been used to this end in the past, [11–16, 19, 22].

The use of surrogates with physiochemical properties more similar to microorganisms may allow simulating transmission events without the need for microbial contamination. Silica nanoparticles with encapsulated DNA (SPED) of known nucleotide sequences have been established by Paunescu et al. in order to create inert surrogate tracers that serve to track the presence and distribution of biological and chemical entities (e.g. wastewater, milk, pesticides) [23–26]. Forming part of daily meals of many people through seafood, grains, vegetables and broadly utilized in the food industry, silica has been acknowledged as “safe” by the U.S. Food and Drug Administration 50 years ago [27]. In medicine, e.g. in medical imaging, silica nanoparticles are equally widely used and investigations of their biological degradability and clearance has been conducted [27].

Thus, we investigated the behaviour of SPED as potential surrogate tracers for microbial transmission pathways in health care. More specifically, we aimed to explore their transmission pattern in patient care processes, to study the consequences of hand hygiene actions on SPED, and to contrast transfer dynamics of SPED with those of *E. coli*.

## Methods

### Silica particles with encapsulated DNA

**Fig. 1:**
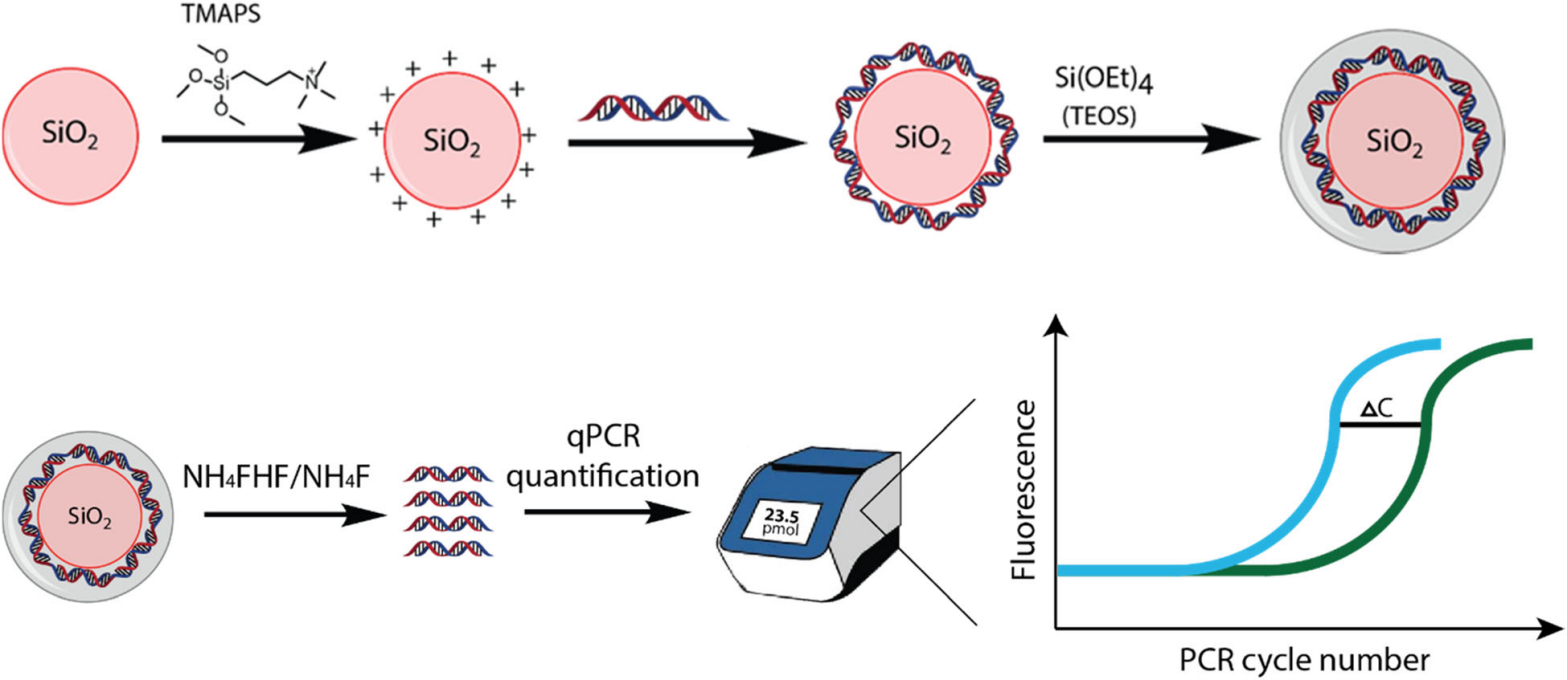
Scheme of synthesis of SPED and analysis with qPCR quantification. Legend: TMAPS, N-trimethoxysilylpropyl-N,N,N- trimethylammonium chloride; TEOS, tetraethyl orthosilicate

The synthesis and characterization of SPED was conducted according to Paunescu et al. [23] (Fig. 1) First, per batch, 4x4ml of silica nanoparticles (50 mg/ml in isopropanol) were surface functionalised in 4 separate falcon tubes by adding 40 μg of N-trimethoxysilylpropyl-N,N,N- trimethylammonium chloride (TMAPS; 50% wt in methanol) and stirred at 900 rounds per minute (rpm). for 12 h at room temperature. The trimethylammonium on the surface gave the silica particles a positive surface charge. For DNA adsorption on the surface a 2 ml batch of corresponding annealed DNA molecules was added to 200 ml ultrapure MilliQ (mQ) water (150 ng dsDNA/μl, Nanodrop). Of the previously TMAPS functionalised particles, 0.4 g were added to the solution and the batch was shaken for 10 s. Subsequently, 4 μl TMAPS were added, the batch was shaken and then sonicated for 20 s. Next, 62.5 μl of tetraethyl orthosilicate (TEOS) (≥99.0%, Aldrich) were added and the batch was shaken for 5 h. In a next step, 10 ml isopropanol and 5.9 ml TEOS were mixed with 484.1 ml mQ water before adding it to the previous mixture. The batch was then stirred at 600 rpm for 4 days. To demonstrate the potential of multi-tracing, three different batches were produced, each containing a unique DNA sequence. The three particle batches named SPED1, SPED2 and SPED3 had a hydrodynamic size of 218 ± 80 nm, 144.6 ± 46 nm and 173.4 ± 82 nm and a DNA loading of 21 μg, 23 μg, and 26 μg dsDNA/mg of particles, respectively. The suspension used for our experiments was prepared by diluting the particles to 0.1 mg/ml or 1 mg/ml in mQ water + 10% glycerine. We used glycerine to increase the probability of transfers in our experiments according to preliminary data showing higher transfer rates in the presence of glycerine (data not shown). Glycerine is viscous and therefore increases the stickiness of the test suspension, simulating the adhesive function of bacterial appendages called pili.

### *Escherichia coli* bacterial culture

Standard procedures were used to cultivate bacterial cultures. *Escherichia coli* was cultured overnight in tryptic soy broth (TSB; Sigma-Aldrich, St. Louis, Missouri, USA). An aliquot of 2 ml of the bacterial cell culture was triple washed by spinning down the *E. coli* culture in a 2 ml microcentrifugation tube (Eppendorf AG, Hamburg, Germany) for 5 min at 3500 rpm. After each cycle, the supernatant TSB was removed, and the bacterial culture resuspended in 1 ml phosphate-buffered saline (PBS). The obtained bacterial concentration was 10^8^ CFU/ml. To prevent further bacterial growth during transfer experiments, microcentrifugation tubes were stored temporarily on ice.

### Study procedures

We conducted three experiments to investigate the behaviour of SPED in a simulated care scenario (Experiment 1), while performing hand hygiene (Experiment 2), and comparing SPED transfer dynamics with those of *E. coli* (Experiment 3).

#### Experiment 1 - SPED transmission characteristics in a simulated care scenario

**Fig. 2:**
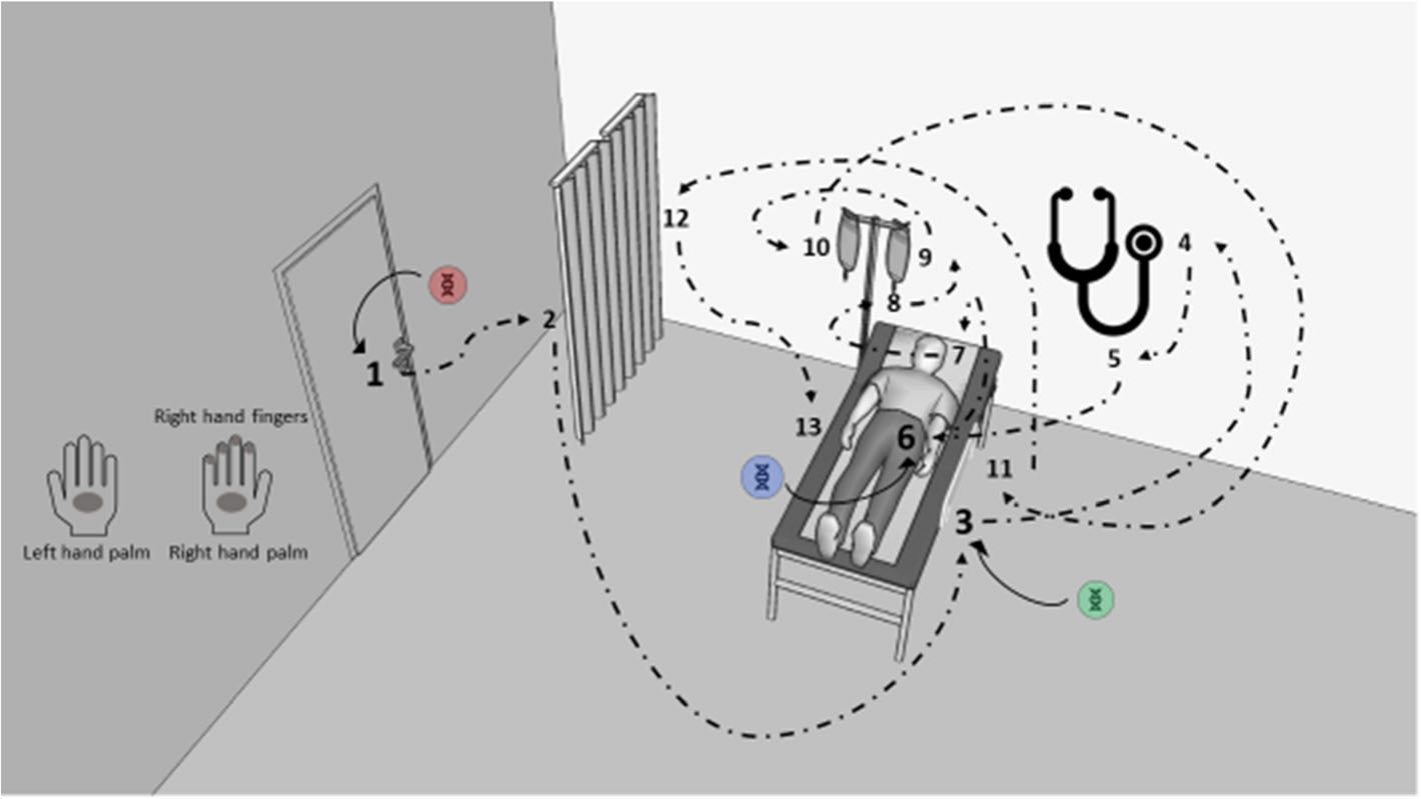
SPED deposition and screening sites in the patient care simulation in Experiment 1. Legend: 1-13, hand-to-surface exposures (1, door handle; 2, privacy screen; 3, bed rail; 4&5, stethoscope; 6, left forearm; 7, eyelid; 8, infusion bag valve; 9&10, infusion bags; 11, bed rail; 12, privacy screen; 13, right forearm. , SPED1 introduction (door handle); , SPED2 introduction (bed rail); , SPED3 introduction (patient forearm); , transmission pathway with numbers representing the consecutive hand-to-surface exposures

We defined a simulated patient care scenario featuring an examination stretcher with bed rails, a privacy screen, and an infusion stand with two infusion bags. A patient actor (henceforth patient) and a HCW trained as a physician (MS; henceforth HCW) conducted the following actions in chronological order: closing a door using the door handle (stainless steel), moving the privacy screen (polyvinylchloride), elevating the bed rail (polypropylene), auscultating the patient’s chest with a stethoscope (polyvinylchloride, PVC), taking the patient’s radial pulse (bare skin), checking pupil reactivity while pulling up the patient’s upper eyelid (bare skin), and changing the infusion bag (polyolefin) (Fig. 2). Two video cameras positioned in a 90° angle on adjacent walls (GoPro® Hero 4 Black edition, GoPro Inc., San Mateo, CA) recorded the scene simultaneously to monitor the sequence.

The scenario featured 13 experimentally pre-defined hand-to-surface exposures, of which 10 were fomites and 3 were patient skin. The sites were chosen according to our observational experience in healthcare settings as being habitual [8]. We marked swabbing areas of 1x3cm on each of the 13 sites. The sites and both hands and fingers of the HCW were swabbed prior to every simulation to demonstrate they were free of SPED. The swabbing technique included with 20% glycerine solution premoistened sterile cotton swabs (Naturaline Wattestäbchen, Steinfels Swiss, Winterthur, Switzerland) that were rolled three times over the area and then stored in a 2 ml microcentrifugation tube at room temperature. This swabbing procedure applied to all following swabs in our experiments.

We inoculated three of the 13 sites with 0.2 ml of the SPED test suspensions (1 mg/ml) and left to air dry for 5 min, namely the door handle with SPED1, the bed rail with SPED2, and the left forearm of the patient with SPED3 (Fig. 2). We decided to use three different initial deposition sites with three SPED with distinct DNA codes to test the parallel application and recovery of distinguishable SPED batches in the same healthcare scenario. Throughout the entire simulated care scenario, the patient was laying on his back on the examination stretcher with his bare arms placed beside him without contact to his body or the examination stretcher. Subsequently, the HCW actor performed the care scenario while exclusively touching the 13 marked sites in a natural way. Once the simulation scenario was concluded, we swabbed the 13 marked test areas as well as both of the HCW’s palms and the fingers of her dominant right hand, resulting in 48 specimens overall. After the experiment, the microcentrifugation tubes were immediately transported to the laboratory for processing.

The experiment was repeated three times on different days and in different simulation rooms, each, referred to as ‘runs’. The repetitions were to add validity to the findings.

#### Experiment 2 - effect of hand hygiene action on SPED concentration

We tested the impact of hand disinfection with alcohol-based handrub and hand washing using soap and water on SPED skin concentration. Three groups of five participants, each, were formed: a control group (CTRL), a hand disinfection group (DISINF), and a hand washing group (WASH). First, swabs were taken from the right thenar of each participant to demonstrate no SPED were present. Subsequently, a previously marked area of 2x2cm on the palmar side of the right thenar of each participant was inoculated with 0.2 ml of the SPED1 test solution (0.1 mg/ml). The fluid was dispensed over the palms of both hands by rubbing both hands against each other for 10 s. Once dry, swabs were taken from the previously marked area of the right thenar of each person, and additionally from the dorsal hypothenar of DISINF participants to test for dispersal by rubbing hands with alcohol-based handrub.

CTRL participants held both their hands in the air without any contact for 1 min. DISINF participants performed hand hygiene according to the norm EN1500 using 3 ml of a commercially available alcohol-based handrub (80% Ethanol with 1% Glycerine, B. Braun Medical AG, Sempach, Luzern, Switzerland). WASH participants washed their hands for 30 s with common soap (Liquid Soap®, Sterisol AB, Vadstena, Sweden) and water using the norm EN1499 procedure. The right thenar of all participants – and additionally the dorsal hypothenar in DISINF participants – were swabbed again after the non-contact waiting time in the CTRL group and hand hygiene actions in the DISINF and WASH group. Thereafter the test tubes were immediately transported to the laboratory for analysis.

#### Experiment 3 – transmission dynamics of SPED compared with *Escherichia coli*

We analysed transmission tendencies of *E. coli* and SPED. Prior to the experiment, a piece of laminate (melamine resin) was rinsed with 70% ethanol, wiped with a sterile cloth and allowed to air dry. Forty series of four squares of 2x2cm were defined. The four squares were marked as origin surface (ORIGS), 1st transfer surface (1TS), 2nd transfer surface (2TS) and control surface (CTRLS), respectively. Next, samples of the surface and gloves (GLOVES) were taken by either rinsing the surface with 100 μl of PBS or by cutting off the glove’s index fingertip and adding it to a 1.5 ml micro-centrifugation tube filled with 100 μl of PBS to prove sterility. Then, 20 μl of the *E. coli* solution was spread onto ORIGS, and with intervals of 2 min, onto CTRLS. The 2-min interval allowed for continuous sampling of surfaces with identical times between surface inoculation and subsequent sampling. A two-step transmission was performed from ORIGS ➔ 1TS ➔ 2TS by pressing the gloved index finger onto the corresponding areas in that order. Samples were taken as described above. The CTRLS served as a reference for the number of *E. coli* or nanoparticles recovered from an inoculated surface in the absence of a transfer event, and so not part of the transfer. This procedure was repeated until there were 20 replicates. The same procedure was reproduced for 20 replicates with 20 μl of the SPED solution (0.1 mg/ml).

### Quantification of SPED

To quantify the amount of DNA particles from each swab after having performed the experiments, the cotton swab was placed in a 2 ml microcentrifugation tube (Eppendorf AG, Hamburg, Germany) with 200 μl of mQ water (Fig. 1). Each sample was ultrasonicated for 1 min and vortexed for 10 s. To release the DNA from the silica coating, 1%vol of a highly diluted solution of buffered oxide etch (BOE) with 0.03 wt% ammonium hydrogen difluorid (NH4FHF, pure, Merck) and 0.02 wt% ammonium fluoride (NH4F, puriss., Sigma-Aldrich, St. Louis, Missouri, USA) were added to the sample. The resulting suspension was then analysed by quantitative PCR (LightCycler® 96) in a multiplex setup with three sequence-specific fluorescent probes (SPED1: Hex, SPED2: Texas Red, SPED3: FAM). All three fluorescent probes could be detected simultaneously and allowed to quantify each particle separately. The qPCR total reaction volume was 12.5 μl consisting of 2.5 μl sample solution, 0.2 μl of each primer (3x forward and reverse),0.125 μl of each probe (Microsynth AG) and Mastermix 2x (GoTaq® Probe, Promega). The qPCR program consisted of a preincubation step for 600 s at 95 °C, followed by a 2-step cycling for 15 s at 95 °C and for 60 s at 56 °C. For quantification, a dilution series with known concentration of particles was performed.

### Sampling of *Escherichia coli*

Once the transfer procedures were completed, the samples were processed within 2 h. For sampling, the areas contaminated with *E. coli* were rinsed with 100 μl of PBS, which was then added to a further quantity of 100 μl of PBS in a 1.5 ml micro-centrifugation tube, and vortexed. As for gloves (Nitrile latex free, LLG Labware, Meckenheim, Germany), the index fingertip was cut off after performing the transmissions, placed in a 1.5 ml micro-centrifugation tube containing 200 μl of PBS and subsequently vortexed. Aliquots of 100 μl of undiluted specimen solution and 100 μl from 1:10–1:100′000 dilutions were plated onto TBX Agar (Sigma-Aldrich, St. Louis, Missouri, USA) and incubated at 36 ± 1 °C for 24 h.

### Statistical analysis

Descriptive analysis of our data in Experiment 1 and 2 was performed with Microsoft® Excel® 2016. To estimate the detection limit of the nanoparticles in our experiments, we used the Method Detection Limit (MDL). The MDL is defined as “the minimum measured concentration of a substance that can be reported with 99% confidence that the measured concentration is distinguishable from method blank results.” [28] Values that are below MDL are considered to be negative, values higher than the limit as positive. The MDL was calculated as the mean concentration of the blank samples plus three times the standard deviation.

For Experiment 3, test statistics including t-test were conducted with R Statistical Software (version 3.6.0, R foundation, Vienna, Austria). We defined transfer percent as the fraction of *E. coli* or SPED recovered from 1TS, 2TS, and glove as a percentage of the seeded inoculum recovered from the surface by swabbing, defined by CTRLS.

## Results

### Experiment 1 - SPED transmission characteristics in a simulated care scenario

**Fig. 3:**
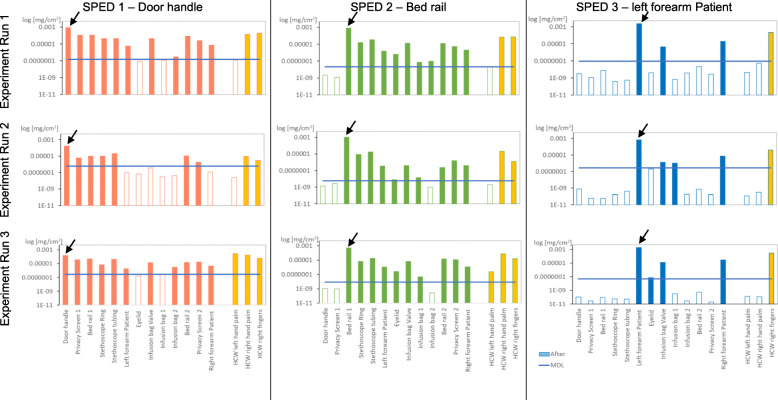
Detection of SPED1–3 on environmental and patient body sites and healthcare worker’s hands in Experiment 1. Legend: *red/green/blue,* 13 test sites of SPED1-3; *yellow,* healthcare workers’ hands; *blue line,* MDL minimal detection limit; , introduction of SPED1, SPED2 and SPED3, respectively

According to the video analysis, the sequence of touched surfaces was maintained as scheduled for all runs. Overall, we collected 96 swabs, each analysed for the three different DNA sequences, resulting in 288 values. All 39 control swabs on the 13 test sites for the three scenario runs before SPED were negative. Equally, none of the swabs tested positive for a given SPED type ahead of its deposition site in the care sequence (Fig. 3). For runs 1, 2 and 3, the MDL determined from the recovered sample for SPED1 was 1.49^− 7^ mg/ml; 3.70^− 7^ mg/ml, 9.95^− 8^ mg/ml, for SPED2, 1.80^− 8^ mg/ml; 5.20^− 9^ mg/ml; 3.75^− 9^ mg/ml and for SPED3 4.24^− 8^ mg/ml, 6.74^− 8^ mg/ml; 1.25^− 8^ mg/ml, respectively. Accordingly, overall, the number of positive results among all sites except the deposition site and hands were 26 of 36 (72.2%) for SPED1, 28 of 30 (93.3%) for SPED2, and 8 of 21 (38.1%) for SPED3. As for hands, right hand fingers revealed positive SPED results in 9 of 9 (100%), right hand palm in 6 of 9 (66.6%) and left-hand palm in 2 of 9 (22.2%). Annex Table 1 shows the proportion of all sites with positive SPED results. Detection rates for the tracers varied among the touch-sites. Consistently positive samples were found on the left and right forearm of the patient, on the infusion bag valve, and on HCW’s fingers. No differences were found in detectability regarding the material on which the test suspension was deposited.

### Experiment 2 - effect of hand hygiene procedure on SPED concentration

**Fig. 4:**
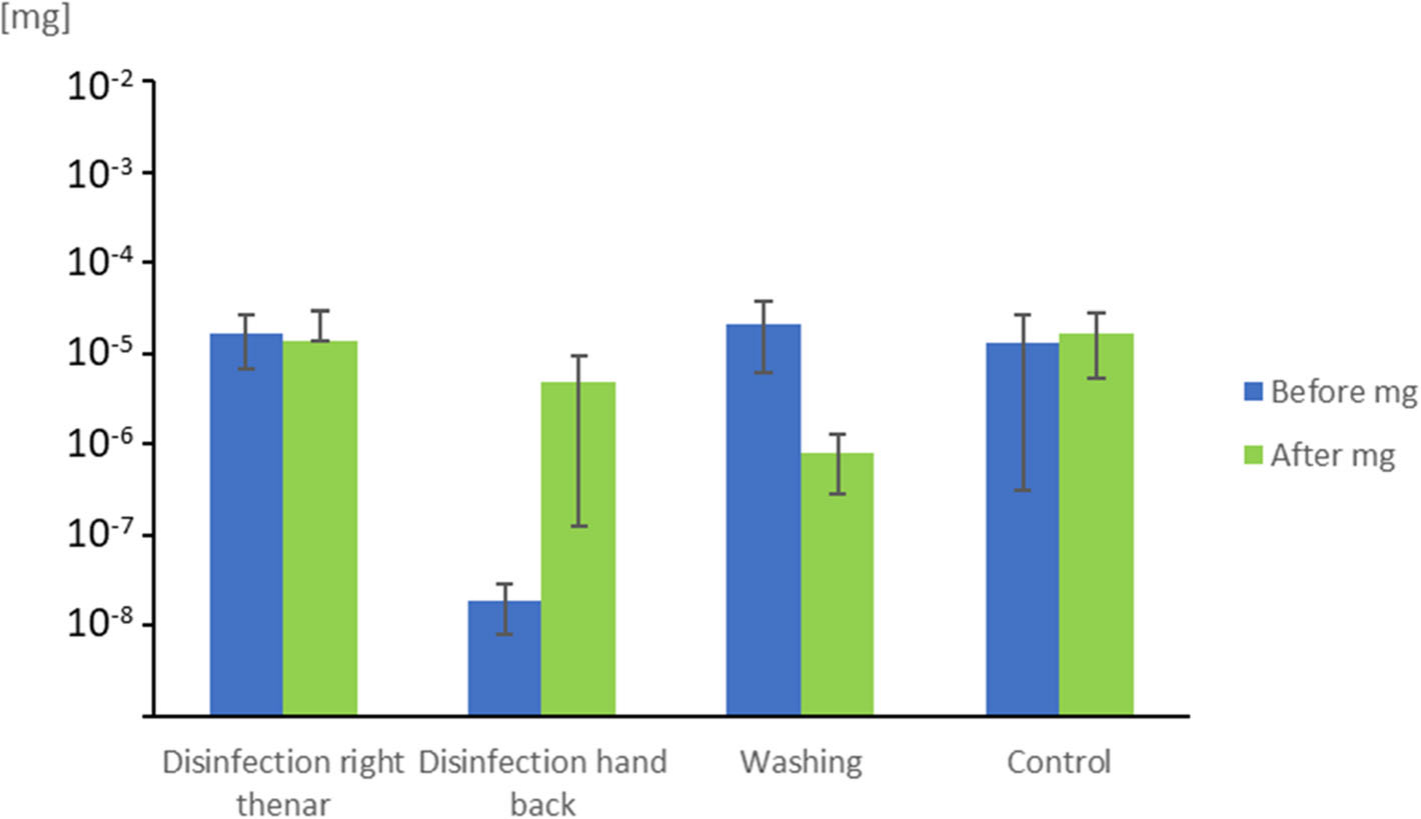
SPED1 skin concentration before and after hand cleaning procedures in Experiment 2. Legend: *Disinfection*, hand cleansing with alcohol-based handrub; *Washing*, washing hands with soap and water; *Control*, no hand hygiene action; Error bars = Standard deviation

All samples taken before applying SPED were negative. The mean SPED1 DNA-concentration before and after the experiment are presented in Annex Table 2. This corresponds to a 24% increase of SPED concentration for CTRL, a reduction of 17% on the thenar and 25,670% increase on the back of the hand for DISINF, and a reduction of 96% for WASH (Fig. 4).

### Experiment 3 – transmission dynamics of SPED compared with *Escherichia coli*

**Fig. 5:**
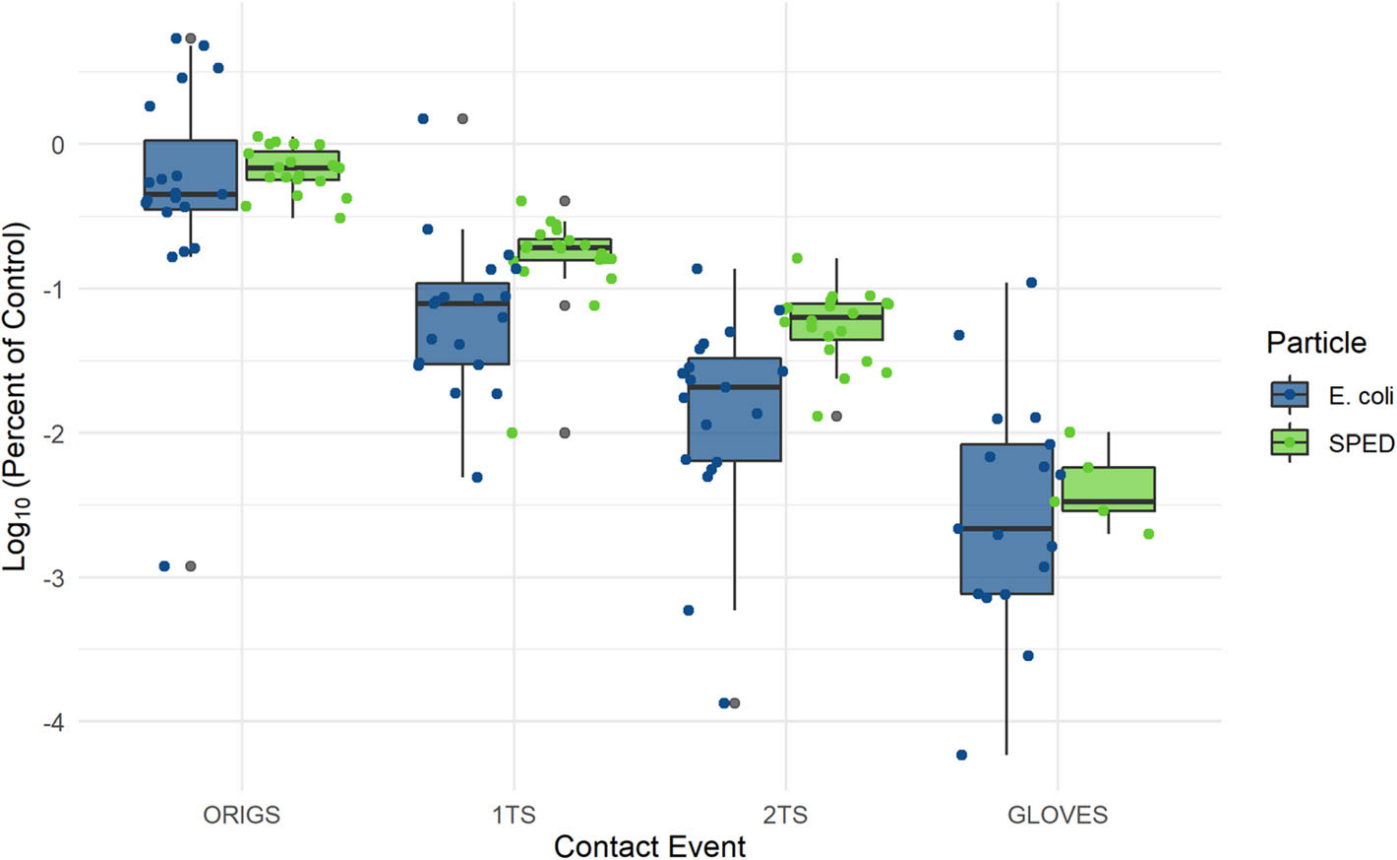
Log10 percent transfers for the original, 1TS, 2TS, and GLOVES in Experiment 3. Legend: ORIGS, origin surface; 1TS, first surface touched after the origin surface; 2TS, surface touched after 1TS; GLOVES, nitrile disposable glove index finger tested after all three surfaces have been touched

In total, 40 two-step transfers were conducted, 20 for *E. coli* and 20 for SPED. Samples taken from gloves and surface prior to the experiment were negative. The results are displayed in Annex Table 3 and Fig. 5.

## Discussion

We explored SPED as a promising agent to trace microbial transmission in healthcare.

First, we were able to establish the suitability of SPED to trace the transmission pathways of microbes in a simulated care scenario featuring a typical sequence of hand-to-surface exposures. SPED concentration showed decreasing trends with consecutive touched surfaces downstream of their deposition site with consistently negative upstream results. This is also the proof that parallel recovery of SPED marked with different DNA can be distinguished when applied in a healthcare environment. Importantly, they also exhibited discriminative quantitative recovery patterns on hands and surfaces reflecting the way objects were touched, i.e. with the whole hand vs with fingertips only. The markers SPED1 – initially deposited on the door handle - and SPED2 – initially deposited on bed rail 1 - were transmitted from their deposit site to the palm of the HCW’s right hand and fingers, and subsequently spread onto almost all surfaces except eyelid and one of the two infusion bags that were manipulated with fingertips. In contrast, the initial deposition site of SPED3 – the left forearm of the patient – was naturally touched by the HCW’s right hand fingers, which resulted in an transmission to and recovery from consecutive contact-sites that were touched by fingers only, i.e. opposite forearm and infusion valve (Fig. 3). These findings suggest that SPED could be used for a detailed understanding of transmission pathways in healthcare settings with high special resolution, especially benefitting from the possibility to use identifiable SPED in parallel and avoid the problem of contamination in case of repeated investigations in the same environment.

Second, in Experiment 2, we found that hand washing with soap and water resulted in a nearly complete removal of SPED from hands and thus ideally simulated the mobilisation and dilution of microorganisms. In contrast, hand cleaning with alcohol-based handrub led to the dispersion of SPED to the back of the hands. This is the main shortcoming of SPED as a surrogate for bacteria and viruses that are destroyed by alcohol-based handrub and effective surface disinfectants. It would be desirable to develop the SPED tracer system further to mitigate this flaw. At best, inertia against disinfectants simulates the behaviour of partially inert microorganisms such as spores (e.g. *Clostridioides difficile)* and non-enveloped virus (e.g. Norovirus) [29, 30]. The persistence of SPED in the environment can be seen as another shortcoming that is, however, counterbalanced by the fact that SPED are marked by known DNA-sequences per batch and thus, can be distinguished with each experiment run.

Third, and most importantly, SPED showed a nearly parallel decrease in concentration on surfaces compared to *E. coli* in the head-to-head two-step transmission Experiment 3. By comparing transmission behaviour of SPED to those of *E. coli* we found similarities in transfer with SPED exhibiting a trend to a lower decrease from one consecutive surface to the other. This suggests that, when used as surrogate markers in real healthcare settings, transmission will be rather over- than underestimated.

Previous studies have evaluated the use of harmless surrogate tracers to simulate contact transmission pathways of potential pathogens in healthcare settings [11–22]. These studies employed four surrogate markers, namely DNA sequences of the cauliflower mosaic virus DNA, [11, 17–21] bacteriophage MS2, a non-pathogenic non-enveloped RNA virus, [11–16] and fluorescent agents [13, 14, 16, 19, 22]. Some of these studies compared fluorescent agents against MS2 bacteriophages [13, 14, 16] or against cauliflower virus DNA [19]; or used the two viral DNA markers in parallel [11]. Only one of these studies compared the behaviour of cauliflower virus DNA and MS2 bacteriophage against *C. difficile* spores and found a similar environmental transmission behaviour, [11] as did we in our comparison between SPED and *E. coli*. Two studies used visible fluorescent and flashlight reflective markers, respectively, as part of an educational intervention on personal protective equipment handling and MS2 bacteriophages as outcome measure [15, 16]. These studies all show an impressively widespread transmission of surrogate markers across the healthcare setting in a short interval of time, even if one considers that their persistence in the environment might be longer than viral or bacterial pathogens.

We used video to verify the correct execution of the experimentally predefined hand-to-surface exposure sequence in a simulated care scenario. One study assessed personal protecive equipment handling quality by video and related it to transmission of MS2 bacteriophage and fluorescent liquid [13]. Two studies outside of healthcare settings estimated the risk of bacterial cross-contamination in farming work with human excreta by combining videography with environmental microbial contamination data into a stochastic-mechanistic simulation of bacterial transmission [31, 32].

In contrast to other methods to investigate pathogen transmission in healthcare settings, using SPED has several advantages. First, silica particles are easy to handle, and their management requires only little specialised expertise. The equipment for the analysis, i.e. qPCR analysers, are common equipment in hospital microbiology laboratories. Second, SPED are widely available, safe, and inexpensive. As an example, the raw materials for three repetitions of the Experiment 1 amounted to ~ 200 USD, including DNA, primers and other reagents. The costs of SPED are mainly determined by the price of the synthetic DNA amplicon, which currently equals 500 USD for 100 mg of newly synthesized DNA. Third, the physical characteristics of SPED, such as electric charge, hydrophobic versus hydrophilic properties, size and weight can be modified to match those of pathogens [33–35]. Forth, and most importantly for a clinical application, the fact that SPED solutions with multiple DNA-codes can be applied and analysed by PCR simultaneously provides the opportunity to study an almost infinite number of scenarios and allows for repeated experiments in the same environment with minimal risks of cross-contamination. As mentioned before, the main limitation is SPED inertia against disinfectants.

This proof of concept study has limitations. The conducted experiments were limited in replications and simulated patient care was a simplified predefined scenario. However, we do not expect a categorically different behaviour of SPED in real-life care settings. The concentration of SPED on consecutive sites was high enough to rise confidence that even much wider dispersal can still be traced. Differences in hand-to-surface exposures, e.g. applied pressure and contact time, in addition to inhomogeneous distribution of the SPED in the test solution and slight variations in the swabbing technique may explain the non-linear decline of recovery rates from consecutive touch sites as well as the variation of positive results in the 3 runs. Future work on SPED tracking should attempt to decrease the technical variation in recovery. Considering that concentrations of pathogens (e.g. Norovirus in faeces) appear to be in the same range [36], our results represent an extreme but not unrealistic scenario. Future dilution series could help to establish concentration that are more congruent to daily healthcare conditions. Finally, given the variation in SPED recovery in our study, future work could be invested in standardising the sampling method.

## Conclusions

This proof-of-concept study is, to our knowledge, the first investigation using SPED as a surrogate tracer to study pathogen transmission in healthcare. Further studies in various real-life care settings – and modifying SPED characteristics to align even better with pathogen features – will show their potential to investigate outbreaks and endemic infectious risks at a system level.

In conclusion, SPED spread quickly over all the touch-sites through hand contact and allow to determine their origin and mimic the transmission of live bacteria. They represent a convenient and safe tool to explore pathogen spread from an infinite number of sites simultaneously. They can be developed further to become a central asset for a more effective infection prevention in healthcare and beyond.

## Supplementary information

**Additional file 1: Table S1.** Number of sites testing positive for SPED among over all three runs in Experiment 1. **Table S2.** Mean SPED1 DNA concentration before and after Experiment 2. **Table S3.** Recovery fraction of SPED and *E. coli* after the two-step transfer in Experiment 3.

## Data Availability

Not applicable. Please contact author for data requests.

## Abbreviations

SPED: Silica nanoparticles with encapsulated DNA
*E. coli*: *Escherichia coli*
HCW: Healthcare worker
TMAPS: N-trimethoxysilylpropyl-N,N,N- trimethylammonium chloride;
TEOS: Tetraethyl orthosilicate
TSB: Tryptic soy broth
PBS: Phosphate-buffered saline
CTRL: Control group in Experiment 2
DISINF: Disinfection group in Experiment 2
WASH: Hand washing group in Experiment 2
ORIGS: Origin in Experiment 3
1TS: First transfer in Experiment 3
2TS: Second transfer in Experiment 3
CTRLS: Control in Experiment 3
BOE: Buffered oxide etch
MDL: Method Detection Limit

## References

[1] ECDC (2015). Annual Epidemiological Report 2014. Antimicrobial resistance and healthcare-associated infections.

[2] Harbarth S, Sax H, Gastmeier P (2003). The preventable proportion of nosocomial infections: an overview of published reports. J Hosp Infect..

[3] WHO (2011). Report on the Endemic Burden of Healthcare-Associated Infection Worldwide.

[4] Pittet D, Allegranzi B, Sax H, Dharan S, Pessoa-Silva CL, Donaldson L (2006). Evidence-based model for hand transmission during patient care and the role of improved practices. Lancet Infect Dis.

[5] Wolfensberger A, Clack L, Kuster SP, Passerini S, Mody L, Chopra V (2018). Transfer of pathogens to and from patients, healthcare providers, and medical devices during care activity-a systematic review and meta-analysis. Infect Control Hosp Epidemiol.

[6] Sax H, Allegranzi B, Uçkay I, Larson E, Boyce J, Pittet D (2007). ‘My five moments for hand hygiene’: a user-centred design approach to understand, train, monitor and report hand hygiene. J Hosp Infect..

[7] Schreiber PW, Sax H, Wolfensberger A, Clack L, Kuster SP (2018). Swissnoso. The preventable proportion of healthcare-associated infections 2005-2016: systematic review and meta-analysis. Infect Control Hosp Epidemiol.

[8] Clack L, Scotoni M, Wolfensberger A, Sax H (2017). “First-person view” of pathogen transmission and hand hygiene - use of a new head-mounted video capture and coding tool. Antimicrob Resist Infect Control.

[9] Ward MA, Schweizer ML, Polgreen PM, Gupta K, Reisinger HS, Perencevich EN (2014). Automated and electronically assisted hand hygiene monitoring systems: a systematic review. Am J Infect Control.

[10] Braun BI, Kusek L, Larson E (2009). Measuring adherence to hand hygiene guidelines: a field survey for examples of effective practices. Am J Infect Control.

[11] Alhmidi H, John A, Mana TC, Koganti S, Cadnum JL, Shelton MB (2017). Evaluation of Viral Surrogate Markers for Study of Pathogen Dissemination During Simulations of Patient Care. Open Forum Infect Dis.

[12] Koganti S, Alhmidi H, Tomas ME, Cadnum JL, Jencson A, Donskey CJ (2016). Evaluation of hospital floors as a potential source of pathogen dissemination using a nonpathogenic virus as a surrogate marker. Infect Control Hosp Epidemiol.

[13] Kwon JH, Burnham CD, Reske KA, Liang SY, Hink T, Wallace MA (2017). Assessment of healthcare worker protocol deviations and self-contamination during personal protective equipment donning and doffing. Infect Control Hosp Epidemiol.

[14] Alhmidi H, Koganti S, Tomas ME, Cadnum JL, Jencson A, Donskey CJ (2016). A pilot study to assess use of fluorescent lotion in patient care simulations to illustrate pathogen dissemination and train personnel in correct use of personal protective equipment. Antimicrob Resist Infect Control.

[15] Tomas ME, Cadnum JL, Mana TS, Jencson AL, Koganti S, Alhmidi H (2016). Utility of a novel reflective marker visualized by flash photography for assessment of personnel contamination during removal of personal protective equipment. Infect Control Hosp Epidemiol.

[16] Tomas ME, Kundrapu S, Thota P, Sunkesula VC, Cadnum JL, Mana TS (2015). Contamination of health care personnel during removal of personal protective equipment. JAMA Intern Med.

[17] Oelberg DG, Joyner SE, Jiang X, Laborde D, Islam MP, Pickering LK (2000). Detection of pathogen transmission in neonatal nurseries using DNA markers as surrogate indicators. Pediatrics..

[18] Jiang X, Dai X, Goldblatt S, Buescher C, Cusack TM, Matson DO (1998). Pathogen transmission in child care settings studied by using a cauliflower virus DNA as a surrogate marker. J Infect Dis.

[19] Alhmidi H, Cadnum JL, Jencson AL, Gweder AA, Donskey CJ (2019). Sharing is not always a good thing: use of a DNA marker to investigate the potential for ward-to-ward dissemination of healthcare-associated pathogens. Infect Control Hosp Epidemiol.

[20] John A, Alhmidi H, Cadnum JL, Jencson AL, Donskey CJ (2017). Contaminated portable equipment is a potential vector for dissemination of pathogens in the intensive care unit. Infect Control Hosp Epidemiol.

[21] Alhmidi H, Koganti S, Cadnum JL, Jencson AL, John A, Donskey CJ (2017). Dissemination of a nonpathogenic viral DNA surrogate marker from high-touch surfaces in rooms of long-term care facility residents. Am J Infect Control.

[22] Birnbach DJ, Rosen LF, Fitzpatrick M, Carling P, Munoz-Price LS (2015). The use of a novel technology to study dynamics of pathogen transmission in the operating room. Anesth Analg.

[23] Paunescu D, Puddu M, Soellner JO, Stoessel PR, Grass RN (2013). Reversible DNA encapsulation in silica to produce ROS-resistant and heat-resistant synthetic DNA ‘fossils’. Nat Protoc.

[24] Grass RN, Schälchli J, Paunescu D, Soellner JOB, Kaegi R, Stark WJ (2014). Tracking trace amounts of submicrometer silica particles in wastewaters and activated sludge using silica-encapsulated DNA barcodes. Environ Sci Technol Letters.

[25] Bloch MS, Paunescu D, Stoessel PR, Mora CA, Stark WJ, Grass RN (2014). Labeling milk along its production chain with DNA encapsulated in silica. J Agric Food Chem.

[26] Mora CA, Schärer H-J, Oberhänsli T, Ludwig M, Stettler R, Stoessel PR (2016). Ultrasensitive quantification of pesticide contamination and drift using silica particles with encapsulated DNA. Environ Sci Technol Letters..

[27] Croissant JG, Fatieiev Y, Khashab NM (2017). Degradability and Clearance of Silicon, Organosilica, Silsesquioxane, Silica Mixed Oxide, and Mesoporous Silica Nanoparticles. Adv Mater.

[28] EPA USEPA (2016). Definition and procedure for the determination of the method detection limit, revision 2.

[29] Tuladhar E, Hazeleger WC, Koopmans M, Zwietering MH, Duizer E, Beumer RR (2015). Reducing viral contamination from finger pads: handwashing is more effective than alcohol-based hand disinfectants. J Hosp Infect.

[30] Jabbar U, Leischner J, Kasper D, Gerber R, Sambol SP, Parada JP (2010). Effectiveness of alcohol-based hand rubs for removal of Clostridium difficile spores from hands. Infect Control Hosp Epidemiol.

[31] Julian TR, Pickering AJ (2015). A pilot study on integrating videography and environmental microbial sampling to model fecal bacterial exposures in Peri-urban Tanzania. PLoS One.

[32] Julian TR, Vithanage HSK, Chua ML, Kuroda M, Pitol AK, Nguyen PHL (2018). High time-resolution simulation of E. coli on hands reveals large variation in microbial exposures amongst Vietnamese farmers using human excreta for agriculture. Sci Total Environ.

[33] Wang Y, Zhao Q, Han N, Bai L, Li J, Liu J (2015). Mesoporous silica nanoparticles in drug delivery and biomedical applications. Nanomedicine..

[34] Hartlen KD, Athanasopoulos AP, Kitaev V (2008). Facile preparation of highly monodisperse small silica spheres (15 to >200 nm) suitable for colloidal templating and formation of ordered arrays. Langmuir..

[35] Bagwe RP, Hilliard LR, Tan W (2006). Surface modification of silica nanoparticles to reduce aggregation and nonspecific binding. Langmuir..

[36] Julian TR (2016). Environmental transmission of diarrheal pathogens in low and middle income countries. Environ Sci Process Impacts.

