# Supplementary material for: Silica nanoparticles with encapsulated DNA (SPED) – a novel surrogate tracer for microbial transmission in healthcare
Source: Antimicrob Resist Infect Control. 2020 Sep 16;9:152. doi: 10.1186/s13756-020-00813-7 (PMC7493369; doi:10.1186/s13756-020-00813-7)

| Annex Table 1. Number of sites testing positive for SPED among over all three runs in Experiment 1 | | | | |
| --- | --- | --- | --- | --- |
|  | **Number of positive sites for SPED1 deposited on**  **door handle** | **Number of positive sites for SPED2**  **deposited on bed rail 1** | **Number of positive sites for SPED3**  **deposited on left patient forearm** | **Overall number of positive sites for SPED1-3 (denominator)** |
| Door handle | **3** | 0* | 0* | 3 (3) |
| Privacy screen 1 | 3 | 0* | 0* | 3 (3) |
| Bed rail 1 | 3 | **3** | 0* | 6 (6) |
| Stethoscope ring | 3 | 3 | 0* | 6 (6) |
| Stethoscope tubing | 3 | 3 | 0* | 6 (6) |
| Left forearm patient | 2 | 3 | **3** | 8 (9) |
| Eyelid | 0 | 3 | 1 | 4 (9) |
| Infusion bag valve | 2 | 3 | 3 | 8 (9) |
| Infusion bag 1 | 0 | 3 | 1 | 4 (9) |
| Infusion bag 2 | 2 | 2 | 0 | 4 (9) |
| Bed rail 2 | 3 | 3 | 0 | 6 (9) |
| Privacy screen 2 | 3 | 3 | 0 | 6 (9) |
| Right forearm patient | 2 | 3 | 3 | 8 (9) |
| Legend: The numbers show the sum of test sites in all three experiment runs that tested positive for SPED1 (deposited on door handle; bold), SPED2 (initially deposited on bed rail 1), SPED3 (initially deposited on left patient forearm), and sum SPED1 to SPED3; e.g. the patient eyelid never tested positive for SPED1 (picked up by the healthcare worker from the door handle) but was positive in all three runs for SPED1 (picked up from bed rail 1) and SPED3 (picked up from left patient forearm), which results a sum of 4 of 9 test sites with positive SPED recovery, overall. * Test sites upstream from the initial deposition site of the corresponding SPED1-3. The number of positive sites at the initial deposition site is marked in bold. | | | | |

| Annex Table 2. Mean SPED1 DNA concentration before and after Experiment 2 | | |
| --- | --- | --- |
|  | **Before** | **After** |
| *DISINF right thenar* | 8.25^-5^mg/ml±4.84^-5^mg/ml | 6.88^-5^±7.60^-5^mg/ml |
| *DISINF hand back* | 9.34^-8^±5.35^-8^mg/ml | 2.41^-5^±2.35^-5^mg/ml |
| *WASH* | 1.1^-4^±7.61^-5^mg/ml | 3.96^-6^±2.52^-6^mg/ml |
| *CTRL* | 6.69^-5^±6.53^-5^mg/ml | 8.32^-5^±5.61^-5^mg/ml |
| Legend: *Disinfection*, hand cleansing with alcohol-based handrub; *Washing*, washing hands with soap and water; *Control*, no hand hygiene action; results represent mean (±standard deviation) | | |

| Annex Table 3. Recovery fraction of SPED and *E. coli* after the two-step transfer in Experiment 3 | | | | |
| --- | --- | --- | --- | --- |
|  | **ORIGS*_E. coli_*** | **1TS*_E. coli_*** | **2TS*_E. coli_*** | **GLOVES*_E. coli_ _(n=20)_*** |
| *Median recovery fraction (interquartile range)* | -0.34 (-0.44; 0.02) | -1.10(-1.52; -0.96) | -1.68 (-2.19; 1.48) | -2.66 (-3.11; 2.07) |
| *Median recovered fraction from ORIGS against CTRLS (range)* | 57% (0.1%-540%) | 8% (0.1%-150%) | 2.1% (0.01%-13%) | 0.4% (0.005%-11%) |
|  | **ORIGS_SPED_** | **1TS_SPED_** | **2TS_SPED_** | **GLOVES_SPED_ _(n=5)_** |
| *Median recovery fraction (interquartile range)* | -0.16 (-0.24;-0.04) | -0.71 (-0.80;-0.65) | -1.19 (-1.35;-1.10) | -2.47 (-2.53;-2.23) |
| *Median recovered fraction from ORIGS against CTRLS (range)* | 70% (28%-287%) | 16% (0.3%-40%) | 5.3% (0.1%-16%) | 0.7% (0.1%-55%) |
| Legend: ORIGS, origin surface; 1TS, first surface touched after the origin surface; 2TS, surface touched after 1TS; GLOVES, nitrile disposable glove index finger tested after all three surfaces have been touched; CTRLS, surface serving as reference | | | | |

**SPED sequences**

All sequences are 65 Basepairs long.

Legend:

pF: Primer forward

pR: Primer reverse

probe = Flurorescent probe

**GM-06-SPED1** (5’)TTATGGGCTCTAAGGATCTCTTCGTTGTCGTTAGGTTCCTGCGTTTTTCGATTCGAGGGTGAGTT(3’)

GM-06-S1_pF ATGGGCTCTAAGGATCTC

GM-06-S1_pR CTCACCCTCGAATCGAA

GM-06-S1_probe ACGCAGGAACCTAACGACAACG

GM-06-SPED2

(5’)TATGCGCCTTTATACTCTTATAGGTATCCTGTTGCTGGCACTTTTTTCTAGCAAAGTCTTCTCCT(3’)

GM-06-S2_pF ATGCGCCTTTATACTCTTA

GM-06-S2_pR GGAGAAGACTTTGCTAGAA

GM-06-S2_probe AAGTGCCAGCAACAGGATACCT

GM-06-SPED3

(5’)TAGCTCGTTCATAGAATCACTTCGCCGTACTCAACGTAGTGGTTTTTGTTTAGCTCAAACAGGTT(3’)

GM-06-S3_pF AGCTCGTTCATAGAATCAC

GM-06-S3_pR ACCTGTTTGAGCTAAACAA

GM-06-S3_probe CGCCGTACTCAACGTAGTG

**Dilution series SPED 1-3**


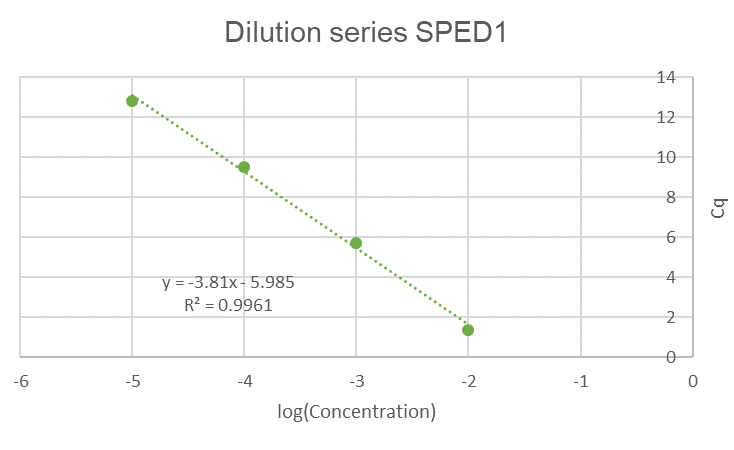


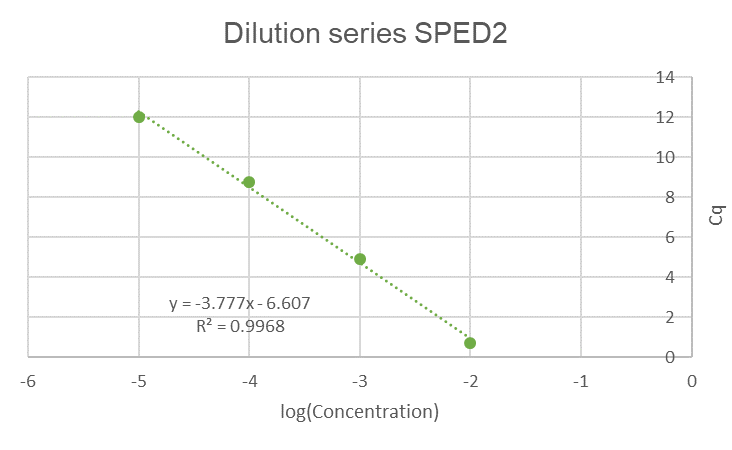


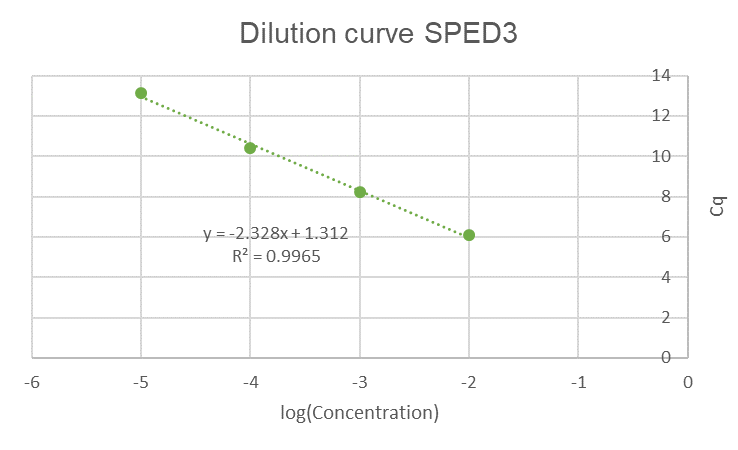

Supplement: Supplementary file 1 — Additional file 1: Table S1. Number of sites testing positive for SPED among over all three runs in Experiment 1. Table S2. Mean SPED1 DNA concentration before and after Experiment 2. Table S3. Recovery fraction of SPED and E. coli after the two-step transfer in Experiment 3. [file 13756_2020_813_MOESM1_ESM.docx]
